# The burden of seasonal influenza in Italy: A systematic review of influenza‐related complications, hospitalizations, and mortality

**DOI:** 10.1111/irv.12925

**Published:** 2021-10-26

**Authors:** Irene Giacchetta, Chiara Primieri, Riccardo Cavalieri, Alexander Domnich, Chiara de Waure

**Affiliations:** ^1^ Department of Medicine and Surgery University of Perugia Perugia Italy; ^2^ Hygiene Unit San Martino Policlinico Hospital ‐ IRCCS for Oncology and Neurosciences Genoa Italy

**Keywords:** burden, complications, influenza, Italy, mortality, systematic review

## Abstract

Reliable country‐specific data on influenza burden play a crucial role in informing prevention and control measures. Our purpose was to provide a comprehensive summary of the available evidence on the burden of seasonal influenza in Italy. We performed a systematic literature review of articles published until July 31, 2020. PubMed, Embase, and Web of Science were searched using terms related to burden, influenza, and Italian population. We included studies investigating seasonal influenza‐related complications, hospitalizations, and/or mortality. Sixteen studies were included: eight (50%) analyzed influenza‐related complications, eight (50%) hospitalizations, and seven (43.8%) influenza‐related deaths. Only three studies (19.7%) concerned pediatric age. The synthesis of results showed that patients with chronic conditions have an increased risk for complications up to almost three times as compared with healthy people. Hospitalizations due to influenza can occur in as much as 5% of infected people depending on the study setting. Excess deaths rates were over sixfold higher in the elderly as compared with the rest of population. Although there are still gaps in existing data, there is evidence of the significant burden that influenza places each year especially on high‐risk groups. These data should be used to inform public health decision‐making.

## 1 INTRODUCTION

Seasonal influenza is an infectious disease that highly affects population health in the Europe.^1^ Worldwide, annual influenza epidemics are estimated to result in about 3 to 5 million cases of severe illness, especially among older adults, young children (<5 years), pregnant women, and individuals with chronic medical conditions.^2^ In high‐income countries, most influenza‐related deaths occur among people aged 65 years or older.^3^ Respiratory complications are the most common sequelae,^4^ and it has been estimated that about 290,000 to 650,000 deaths from respiratory causes^5^, ^6^ and 99,000 to 200,000 deaths from lower respiratory tract infections (LRTIs) are attributable to influenza annually.^7^ Furthermore, several extra‐respiratory complications, such as cardiovascular and nervous system play an important role.^8^ Worryingly, seasonal influenza generally represents an underappreciated public health problem with significant socio‐economic implications.^9^

The monitoring and surveillance of seasonal influenza is possible through data collection and sharing systems, such as FluView in the United States (www.cdc.gov/flu/weekly) and FluNews in Europe (www.flunewseurope.org), that systematically collect data on seasonal influenza and publish periodic reports to inform on epidemiological trends. InfluNet is the Italian nationwide sentinel surveillance system for influenza, coordinated by the Italian National Institute of Health. It collects epidemiological (InfluNet‐Epi) and virological (InfluNet‐Vir) data that are weekly published on FluNews‐Italy (https://www.epicentro.iss.it/influenza/FluNews) reports and uploaded into the European database coordinated by the European Centre for Disease Prevention and Control (ECDC). FluNews‐Italy also integrates findings from other surveillance systems, such as the monitoring system of severe and complicated laboratory‐confirmed cases of influenza, daily mortality among the elderly and InfluWeb (a web‐based surveillance system of influenza‐like illness [ILI]). More information on influenza burden (e.g., complications and hospitalizations) can be gathered from other sources, such as Health for All database (https://www.istat.it/it/archivio/14562) or published papers. However, the available evidence is still suboptimal. For instance, health technology assessment (HTA) projects of different preventive interventions against influenza have brought to light the need for more data.^10^, ^11^, ^12^, ^13^, ^14^

In sum, reliable country‐specific data on influenza burden play a crucial role in informing the planning of prevention and control measures to limit the spread of the disease and minimize associated costs. For this reason, in scientific literature, there are some country specific reviews aiming to assess influenza incidence and clinical and economic burden. The published reviews focus either on a specific geographical area, such as Latin America, sub‐Saharan Africa, Japan, or West Europe, or on a specific age range, such as elderly or pediatric age.^15^, ^16^, ^17^, ^18^, ^19^, ^20^, ^21^ To the best of our knowledge, no review has focused on Italy. Consequently, the present study aims to provide an overview of available data on the burden of seasonal influenza in Italy. Alongside the above‐described Italian databases, this comprehensive review may be of aid for policy makers, health economists, public health practitioners, and other relevant stakeholders.

## 2 MATERIALS AND METHODS

This systematic review of the literature (PROSPERO registration number: CRD42021272644) was conducted following the 2020 PRISMA guidelines (Appendix A).

### 2.1 Identification of eligible studies

All studies quantifying the burden of seasonal influenza in Italy were potentially eligible, independently by initial influenza clinical presentation. The study outcome was the burden of influenza defined here as influenza‐attributable complications, hospitalizations, or deaths. The study population was the entire Italian population, independently of age, health status, and any other variable affected by both laboratory‐confirmed influenza and clinical proxies (e.g., ILI). No formal limits were established for study design. By contrast, the following exclusion criteria were applied: (i) studies evaluating the burden of pandemic influenza; (ii) case reports and case series with no possibility to establish the population burden of influenza; (iii) economic modeling with no original data; (iv) narrative reviews and other forms of the second‐hand research; (v) original studies focusing only on epidemiological and/or virological surveillance of the laboratory‐confirmed influenza and/or ILI.

The literature search was performed by consulting three databases, namely, PubMed, Web of Science (WoS), and Embase. The following search string was used on PubMed: “(epidemiology OR epidemiological OR virolog* OR surveillance OR incidence OR (“attack” AND rate) OR complicat* OR hospitalization OR (inpatient AND (admission OR care)) OR (outpatient AND (admission OR care)) OR (hospital AND (admission OR care OR discharge)) OR ambulatory OR mortality OR death OR sequelae OR visit) AND (influenza OR flu) AND (Italy OR italian)”; this spelling was then adapted to WoS and Embase. No search restrictions were applied. The search was updated to July 31, 2020.

After removing duplicates, papers were screened by title and abstract first. Clearly ineligible studies were discarded. The remaining records were assessed in the full‐text modality.

### 2.2 Data extraction and synthesis

From the articles definitively included in the literature review, the following information were extracted: bibliographic record, study location, study setting (i.e., outpatient, inpatient, institutionalized, and mixed), main demographic characteristics of the study population (e.g., sample size, age, and sex distribution); study period/influenza season, type of outcomes, and their occurrence. Moreover, if available, data were stratified by viral (sub)type and type of outcome.

A meta‐analysis of data was not planned because of the expected heterogeneity in study populations and endpoints. Indeed, data were summarized in a narrative way.

### 2.3 Quality assessment

The Newcastle‐Ottawa Scale (NOS) was used for evaluating the quality of included studies. NOS adopts a star system, with a total score ranging from 0 to 9 and a score ≥7 indicating a high‐quality study. Two investigators separately performed the quality evaluation of each study, and disagreements were settled by a joint re‐evaluation of the original article with a third author. No study was excluded based on quality criteria.

## 3 RESULTS

**FIGURE 1:**
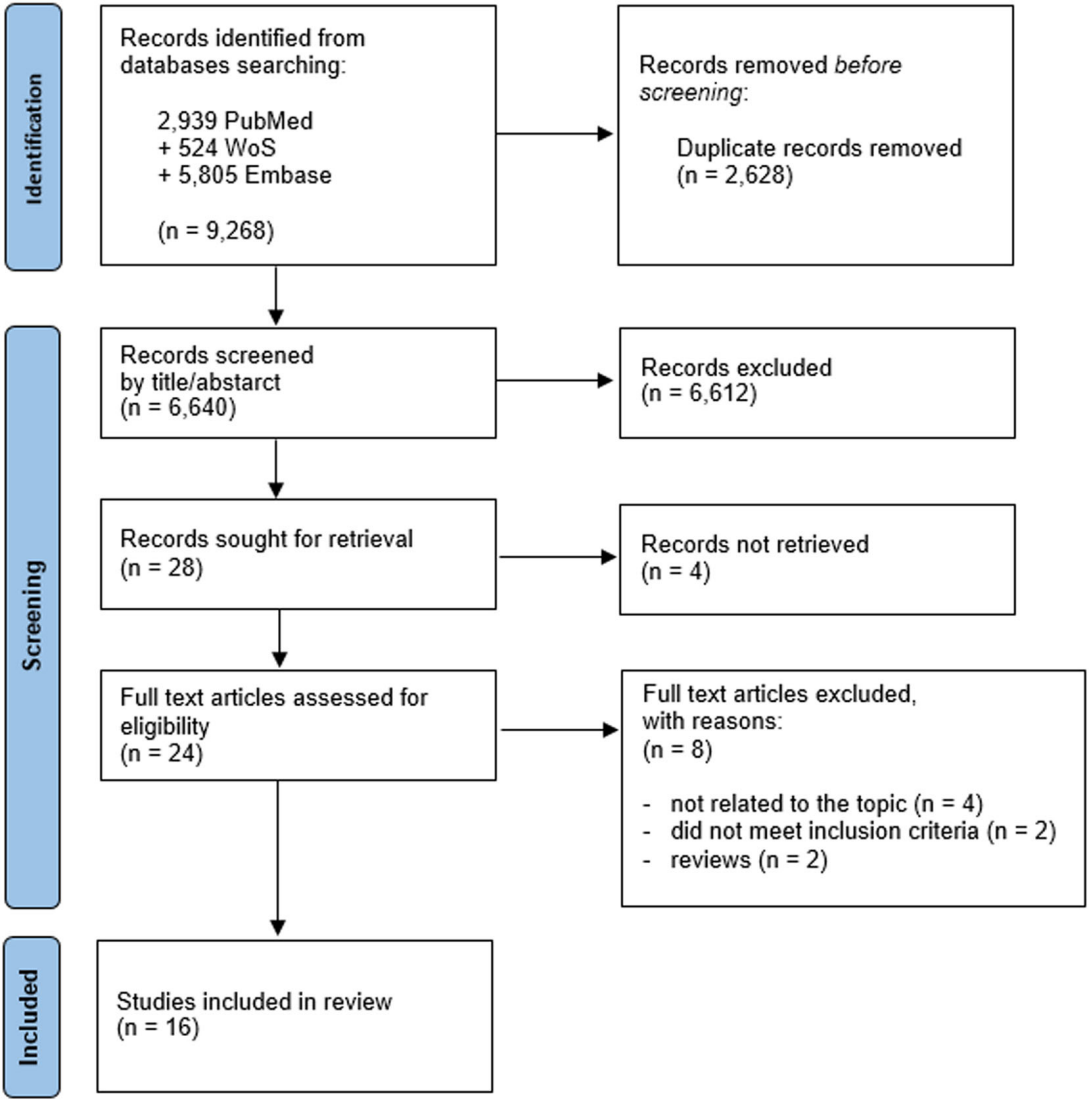
Flow chart of the selection process

The search of the three databases yielded a total of 9268 articles. After duplicates removal, 6640 articles were screened for title and abstract and 28 were selected for full text screening. It was not possible to retrieve four articles. Twenty‐four articles were then screened by full text, and eight studies were excluded with the following reasons: did not meet the inclusion criteria (*n* = 2), not related to the topic (*n* = 4), reviews (=2). Eventually, 16 articles^22^, ^23^, ^24^, ^25^, ^26^, ^27^, ^28^, ^29^, ^30^, ^31^, ^32^, ^33^ published from 2001 to 2020 were included in the qualitative synthesis. Details about the study selection process are shown in the flowchart (Figure 1).

### 3.1 Studies' characteristics and quality

Seven studies (43.7%) were conducted in the Northern Italy,^22^, ^23^, ^25^, ^27^, ^28^, ^30^, ^33^ three (19.7%) in Central Italy,^26^, ^31^, ^32^ and one in Southern Italy.^29^ Four (25%) were nationwide studies^24^, ^34^, ^35^, ^36^ and one (6.2%) was conducted both in Lombardy (in Northern Italy) and Apulia (in Southern Italy).^37^

Five studies (31.2%) were set into a hospital,^22^, ^25^, ^27^, ^32^, ^33^ one (6.2%) in a residential drug‐rehabilitation community,^26^ six (37.5%) in a primary care setting,^23^, ^28^, ^29^, ^30^, ^31^, ^37^ and four (25%) were based on administrative health data.^24^, ^34^, ^35^, ^36^ Regarding studies set in a primary care setting, one (16.67%) was set into a health care unit,^33^ one (16.67%) in the district of a local health authority (LHA),^30^ and four (66.67%) based on general practitioners (GPs) or primary care pediatricians (PCPs).^28^, ^29^, ^31^, ^37^

Three articles (19.7%) concerned the pediatric age,^27^, ^28^, ^32^ three articles (18.7%) concerned adulthood,^22^, ^26^, ^37^ and four (25%) old age,^25^, ^30^, ^31^, ^33^ whereas four (25%) articles referred to the entire population without age distinction.^24^, ^34^, ^35^, ^36^

Eight studies reported data on only one season^22^, ^26^, ^27^, ^28^, ^29^, ^31^, ^33^, ^37^; Rizzo et al reported data from 1970 to 2001,^34^, ^35^ and Bertolani et al reported data from 2008 to 2015.^24^ The other studies reported data on a period of up to four seasons.^23^, ^24^, ^30^, ^32^, ^36^ The characteristics of included studies are reported in Table 1.

**TABLE 1:** Studies' characteristics and data

First author, year	City	Setting	Study population	Period/influenza season	Mean age	Male gender	No. of participants	Outcome (% calculated on the number of participants)		
								No. deaths	No. hospitalizations	No. complications
Bassetti M, 2019	Udine	Hospital	Patients admitted with laboratory‐confirmed influenza	2017/18	48 (range 0–87)	51.7%	29	7 (24.1%)	N.A.	19 (65.5%)
Bellino S, 2019	Treviso	Primary care (Local Health Unit) and hospital	Three cohorts of elderly subjects 65+	2014/15–2016/17	N.A.	43.5–44%	249,005 person‐year (125,253 for vaccinated, and 123,752 for unvaccinated)	4855 and 3276 in vaccinated and unvaccinated subjects	18,355 (11,712 and 6643 in vaccinated and unvaccinated subjects) (ICD‐9‐CM 487, 480–486, 460–466, 490–496, 500–508, 510–516, 410, 422, 427, 428, in primary or secondary diagnosis)	N.A.
Boattini M, 2020	Torino, Lisbon, Nicosia (Cyprus)	Hospitals	Oldest‐old (>85) patients admitted for laboratory‐confirmed influenza and/or RSV infection or developing it during the course of admission for other causes	2017/18–2018/19	89.4 (±3.9 SD)	31.5%	251	35 (13.9%)	N.A.	81 (32.27%) (radiological signs of pneumonia)
Boschini A, 2006	Rimini	Residential drug‐rehabilitation community	Former injecting drug users with ILI	Feb–Mar 2004	N.A.	82.29%	209	N.A.	N.A.	41 (19.62%)
Bosis S, 2005	Milan	Hospital	Children <15 attending the emergency room	Nov 1, 2002‐Mar 31, 2003	4.0 (± 3.7 SD)	50.7%	223 (influenza positive)	N.A.	12 (5.4%)	99 (44.39%)
Esposito S, 2011	Northern Italy	Primary care (PCPs)	Healthy children <14 years of age without severe chronic medical condition but with signs and/or symptoms of ILI	Nov 1, 2008‐Apr 30, 2009	3.8 (± 2 SD)	51%	2143 (influenza positive)	N.A.	16 (0.7%)	424 (19.78%)
Loconsole D, 2019	Apulia region	Primary care (sentinel‐physician network of PCPs and GPs) and hospitals	Patients with ILI, patients hospitalized with severe acute respiratory illness, patients admitted to all ICUs of the region with ARDS	2017/18	N.A.	N.A.	565 (influenza positive)	23 (4.07%) (deaths occurred in patients with ICU‐ARDS)	50 (8.85%) (ICU‐ARDS hospitalizations)	N.A.
Mannino S, 2013	Cremona, Bergamo, Mantova, Lecco, Pavia	Primary care (Local health authorities' district) and hospital	Residents 65+ who sought influenza vaccination (excluding those in hospital, nursing homes or rehabilitation centers)	2006/07–2008/09	76.5 among aTIV; 74.9 among TIV	43.2%	aTIV: 84,665 person‐season, TIV: 79,589 person‐season	N.A.	aTIV: 114 (0.135%), TIV:111 (0.139%) (ICD‐9‐CM 480–487)	N.A.
Manzoli L, 2009	Chieti, Pescara	Primary Care (GPs)	Elderly assisted by GPs	First semester 2007	75.8 (± 7.4 SD)	43.4%	32,457	N.A.	142 (0.44%) (ICD‐9‐CM 480–487)	N.A.
Mastrolia M, 2019	Florence	Hospital	Children 1 month to 14 years with laboratory‐confirmed influenza associated to neurological disease	2017/18–2018/19	27 months (IQR 7–48)	26.67%	15	N.A.	N.A.	1 (6.7%) (impairment in motor skills)
Mikulska M, 2013	Genova	Hospital (HSCT unit)	Adult outpatients seen at least once a month in the HSCT unit	Jan 1 to Mar 31, 2011	N.A.	50%	20 (influenza positive)	N.A.	N.A.	1 (5%) (Clinical and radiological pneumonia)
Sessa, 2001	Lombardy and Puglia	Primary care (GPs)	Patients visited for clinical influenza	Dec 15, 1998, to Mar 15, 1999	40	49.9%	6057	N.A.	26 (0.43%)	2125 (35.1%)
Bertolani A, 2018	Nationwide	N.A.	General population	2008, 2010–2015	N.A.	N.A.	N.A.	N.A.	Average annual number of hospitalizations: 17,488 (3508 observed 487 codes + 13,980 estimated from other codes) (ICD‐9‐CM 422, 427, 428, 460–466, 481–486, 487, 481–486, 490–496, 500–508, 510–516)	N.A.
Rizzo C, 2006	Nationwide	N.A.	General population	1970–2001	N.A.	N.A.	N.A.	Excess mortality rate (per 100,000), P&I and AC respectively: 0.72 and 5.60 age 45–64; 14.13 and 98.86 age >65 (ICD‐8 codes 480–486 and 470–474, ICD‐9480–486 and 487)	N.A.	N.A.
Rizzo C, 2007	Nationwide	N.A.	General population	1970–2001	N.A.	N.A.	N.A.	Excess deaths attributable to influenza: 57,234 from P&I, 318,806 from AC Excess mortality rate (per 100,000), P&I and AC respectively (age‐adjusted): 1.9–2.2 and 11.6–18.6 all ages; 0.4–0.7 and 4.3–6.6 age 45–64; 12.7–14.2 and 71.2–115.7 age 65 + (ICD‐8 codes 480–486 and 470–474, ICD‐9480–486 and 487)	N.A.	N.A.
Rosano, 2019	Nationwide	N.A.	General population	2013/14–2016/17	N.A.	N.A.	5,290,000 estimated ILI	Excess deaths attributable to influenza: 68,068 (1.29% of ILI) Excess mortality rate (per 100,000): 11.6–41.2 all ages; 65.0–147.3 (65+)	N.A.	N.A.

Abbreviations: AC, all cause; ARDS, acute respiratory distress syndrome; GP, general practitioner; HSCT, hematopoietic stem cell transplantation; ICU, intensive care unit; ILI, influenza‐like illness; N.A., not available; PCP, primary care pediatrician; P&I, pneumonia and influenza; RSV, Respiratory Syncytial Virus; SD, standard deviation; TIV, trivalent inactivated vaccine; aTIV, adjuvanted trivalent inactivated vaccine.

The quality of studies varied in the range from 4 to 6 stars (median: 4; mean: 4.562) (Table 2). All the studies were judged to have a representative exposed cohort and a follow up long enough for outcomes to occur. The quality assessment was penalized by the absence of the non‐exposed cohort that prevented assigning three stars for all the studies.

**TABLE 2:** Quality of included studies

Author, year	Representativeness of the exposed cohort	Selection of the unexposed cohort from the same community as the exposed	Ascertainment of exposure: secure record	Outcome of interest not present at start of study	Comparability of cohorts (on the basis of the outcome). Control for:		Assessment of outcome	Follow‐up long enough for outcomes to occur	Adequacy of follow up of cohorts
					Important factor	Additional factors			
Bassetti., 2019	*	N.A.	*		N.A.	N.A.	*	*	
Bellino, 2019	*	N.A.	*		N.A.	N.A.	*	*	
Bertolani, 2018	*	N.A.	*		N.A.	N.A.	*	*	
Boattini, 2020	*	N.A.	*		N.A.	N.A.	*	*	
Boschini, 2006	*	N.A.		*	N.A.	N.A.		*	*
Bosis, 2004	*	N.A.	*	*	N.A.	N.A.	*	*	*
Esposito, 2011	*	N.A.	*	*	N.A.	N.A.	*	*	*
Loconsole, 2019	*	N.A.	*		N.A.	N.A.	*	*	
Mannino, 2012	*	N.A.		*	N.A.	N.A.	*	*	*
Manzoli, 2009	*	N.A.	*	*	N.A.	N.A.	*	*	*
Mastrolia, 2019	*	N.A.	*		N.A.	N.A.	*	*	
Mikulska, 2014	*	N.A.	*	*	N.A.	N.A.	*	*	*
Rizzo, 2006	*	N.A.	*		N.A.	N.A.	*	*	
Rizzo, 2007	*	N.A.	*		N.A.	N.A.	*	*	
Rosano, 2019	*	N.A.	*		N.A.	N.A.	*	*	
Sessa, 2001	*	N.A.		*	N.A.	N.A.		*	*

*Note*: Referring to the Newcastle Ottawa Scale, the star is allocated if methods adopted are considered acceptable.

Abbreviation: N.A., not available.

### 3.2 Health burden of influenza

#### 3.2.1 Complications

Eight articles (50%) evaluated influenza‐related complications^22^, ^25^, ^26^, ^27^, ^28^, ^32^, ^33^, ^37^; all of them analyzed respiratory complications, whereas five articles also analyzed non‐respiratory ones.^26^, ^27^, ^28^, ^32^, ^37^

In the general population, complications occurred in 35.1% of patients visited by GPs for clinical influenza; elderly and patients with concomitant chronic diseases had a significant increased risk (OR, respectively, of 1.7 and 2.9).^37^ According to the study setting, the percentage of people incurring complications fluctuated between 19.6%^26^ and 65.5% in adulthood,^22^ 19.8%^28^ and 44.4% in pediatric age,^27^ and 32.3%^25^ and 57.8%^37^ in the elderly. Fluctuations were due to both the severity of the disease and the study setting (hospital as compared to primary care). In particular, studies performed at the hospital setting^22^, ^25^, ^27^ released higher estimates.

Respiratory complications were the most frequently described and, in the general adult population, bronchitis and pneumonia accounted for 43.2% of complications.^37^ As far as pneumonia is concerned, this occurred in 1.4% of people with clinical influenza visited by GPs^32^ but in 5% of outpatients who underwent hematopoietic stem cell transplantation and former drug users.^26^, ^32^ In the pediatric age, 0.4%–8.1% of children develop pneumonia.^27^, ^28^ Also, non‐respiratory complications, such as cardiac and neurological, were reported in 6.8% and 3.4% out of 29 patients admitted to hospital with severe influenza.^22^ Nevertheless, the frequency of other complications was lower in the other studies.^26^, ^37^ Acute otitis media was mostly described in children and occurred in a percentage ranging from 10.8% and 13.9% of patients.^27^, ^28^

#### 3.2.2 Hospitalizations

Eight articles (50%) evaluated influenza‐related hospitalizations.^23^, ^24^, ^27^, ^28^, ^29^, ^30^, ^31^, ^37^

In the general population, hospitalization occurred in 0.43% of patients visited by GPs for clinical influenza, mostly (76.9%) in at‐risk patients; pneumonia and bronchitis were the most reported causes of hospitalization.^37^ Influenza‐related hospitalizations in pediatric population occurred in 0.7% out of 2143 healthy children without severe chronic medical condition^28^ and in 5.4% of children attending the emergency room.^27^

Loconsole et al^29^ detected 8.85% hospitalizations in intensive care unit (ICU) for acute respiratory distress syndrome (ARDS) among people with a laboratory‐confirmed diagnosis of influenza in Apulia region in the 2017/18 season, but it should be observed that this percentage refer to the subgroup of patients with influenza‐like illness tested for influenza viruses because either hospitalized or for surveillance purpose. Eighty‐four percent of these people were not vaccinated. Another interesting information related to vaccination comes from Bellino et al^23^ that demonstrated a 34%, 22%, 14%, and 12% reduction in hospitalization rates for influenza, pneumonia, respiratory causes, and cardiovascular diseases in vaccinated people in respect to unvaccinated. Mannino et al^30^ detected a very small number of hospitalizations in vaccinated people (<0.2%) and Manzoli et al found even lower hospitalization rates in the elderly population.^31^ These last two studies relied on the consultation of hospital administrative databases and looked only at admissions for influenza and/or pneumonia. Bertolani et al^24^ pointed out an underestimate of influenza‐related hospital admissions, estimating an average of 15,206 hospital admissions for respiratory and cardiovascular complications of influenza in addition to the 4407 admissions reporting influenza specific codes during influenza seasons from 2008/09 to 2014/15.

#### 3.2.3 Mortality

Four articles (25%) evaluated the number of deaths due to influenza in the study population.^22^, ^23^, ^25^, ^29^ Death occurred in 4.1% of patients with laboratory‐confirmed influenza,^29^ but in a higher percentage of patients with severe influenza or ARDS, namely 24.1% and 46% of cases.^22^, ^29^ Death occurred in 13.9% of hospitalized oldest‐old patients with laboratory‐confirmed influenza and/or respiratory syncytial virus infection.^25^ The risk of death was decreased by 33%–39% by vaccination.^23^

Three articles (18.7%) assessed nationwide excess deaths attributable to influenza.^34^, ^35^, ^36^ During the 1970–2001 period, estimated excess influenza‐related mortality rates were 1.9–2.2 per 100,000 considering deaths caused by pneumonia and influenza and 11.6–18.6 per 100,000 considering deaths caused by all causes.^35^ During the same period, the age‐adjusted excess deaths rates in the elderly were 13.3 per 100,000 for pneumonia and influenza and 91.1 per 100,000 for all causes.^34^ For the seasons from 2013/14 to 2016/17, excess influenza‐related mortality rates estimated using the FluMOMO algorithm based on weekly influenza activity and environmental temperature ranged from 11.6 to 41.2 per 100,000 in the general population and from 65.0 to 147.3 per 100,000 in the elderly.^36^

### 3.3 Viral strains contribution

Seven articles (43.8%) analyzed the contribution of viral strains^22^, ^25^, ^28^, ^29^, ^34^, ^35^, ^36^ correlating the complications or the mortality to them (Table 3). In detail, one article evaluated the number of hospitalized and complicated cases of influenza A and B in the pediatric population,^28^ one the number of ARDS hospitalization in ICU caused by influenza A or B,^29^ one the number of complicated patients requiring non‐invasive ventilation (NIV),^25^ three articles evaluated the excess deaths in relation to viral strain^34^, ^35^, ^36^ and one influenza strain found in dead people.^22^ As for complications, heterogeneous results emerged with B strain associated to a higher risk of NIV^20^ and a higher percentage of hospitalization.^28^ About excess mortality, there was evidence of a higher burden of A(H3N2).^34^, ^35^, ^36^

**TABLE 3:** Data on the contribution of influenza strains

First author, year	Data stratified by influenza strain (% calculated on the total of influenza cases by strain)		
	Deaths	Hospitalizations	Complications
Bassetti M, 2019	**B**: 4 (21.1%)	N.A.	N.A.
Boattini M, 2020	N.A.	N.A.	**B**: associated with NIV (OR 3.77; p = 0.041)
Esposito S, 2011	N.A.	**A**: 14 (0.8%); **B**:2 (51%)	**A**: Acute otitis media 195 (11.1%); Acute bronchitis 135 (7.7%), Wheezing 12 (0.7%), Pneumonia 8 (0.5%); **B**: Acute otitis media 36 (9.2%); Acute bronchitis 34 (8.7%), Wheezing 3 (0.8%), Pneumonia 1 (0.3%)
Loconsole D,2019	N.A.	N.A.	**A/H1N1pdm09**: ICU‐ARDS 25 (15.2%); **A/H3N2**: ICU‐ARDS 1 (0.6%); **B**: ICU‐ARDS 24 (6%)
Rizzo C, 2006	**A(H3N2)**: Excess mortality rate P&I 1.04 age 45–64/19.37 age 65+; Excess mortality rate AC 7.53 age 45–64/127.69 age 65+	N.A.	N.A.
Rizzo C, 2007	**A(H3N2)**: excess deaths from AC and P&I four time higher than that for the seasons in which viruses A(H1N1) or B were predominant	N.A.	N.A.
Rosano, 2019	**A(H3N2)**: Remarkable excess death attributable it in seasons 2014–15 and 2016–17	N.A.	N.A.

Abbreviations: AC, all cause; ARDS, acute respiratory distress syndrome; ICU, intensive care unit; ILI, influenza‐like illness; N.A., not available; NIV, Non Invasive Ventilation; P&I, pneumonia and influenza.

## 4 DISCUSSION

This systematic review provides a comprehensive summary of the available scientific literature on the health burden of seasonal influenza in the Italian population. Seven of the 16 papers that we identified were published within the last 3 years (2018–2020), suggesting an expanding interest in the topic.

As expected, respiratory complications were the most frequently described sequalae of the infection, but also non‐respiratory cardiac and neurological complications were reported. On the contrary of other published systematic reviews, we extracted data on the total range of complications, independently by hospitalizations. In this regard, the studies performed at the primary care level in both pediatric and adult population^28^, ^37^ provided a very relevant information on the type and frequency of influenza‐related complications and allowed us to collect data also on those conditions that generally do not determine hospitalization, such as bronchitis and otitis. In particular bronchitis and pneumonia represented approximately half of the complications observed in adult population with clinical influenza at primary care level.^37^ Pneumonia affected a minor percentage of people but occurred in around 5% of individuals at risk.^26^, ^33^ As for the pediatric population, consistent with another systematic review on the topic, we found a lower probability of pneumonia in primary care‐based studies as compared with hospital ones, but we were able to get a more precise estimation of the frequency of otitis media.^15^

The findings of the papers included in this systematic review also showed a significant increased risk for complications among elderly (65+) and patients with at least one chronic condition.^29^, ^37^ This result is aligned with other systematic reviews on the topic.^17^, ^21^

Influenza‐related hospitalizations were shown to be as low as less than 0.1% to more than 5% according to the study setting. Considering the amount of influenza cases occurring each year, we should keep in mind that these results could translate to tens of thousands of hospitalizations each year. Interestingly, hospitalizations were shown to occur in a similar percentage of cases also in the pediatric population. Actually, the Italian Health For All database allows to ascertain that hospitalization rate for influenza and pneumonia in children less than 14 years old was the second highest after hospitalization rate of elderly across different age groups, and this was also confirmed in other countries.^21^ Furthermore, another systematic review attributed 5%–16% of pediatric respiratory hospitalizations to influenza.^19^

In respect to mortality, the findings of this systematic review suggests that influenza is responsible for a relevant excess in mortality rate. Excess death rates for elderly were estimated to be over six times higher than in general population with the most of influenza‐related deaths (65%–96%) occurring in persons 65+.^34^, ^36^ These data were also confirmed by other systematic reviews.^17^, ^38^

It is well‐known that influenza is usually underreported on both death certificates and hospital discharge records either because secondary bacterial co‐infections can develop or because influenza can make some chronic illnesses worse, and this information can be eventually registered as death cause in the place of influenza. Furthermore, it should be noted that patients with influenza‐related complications are not always tested for influenza viruses, or they seek medical care late for influenza virus to be detected from respiratory samples. Indeed, both hospitalizations and deaths due to laboratory‐confirmed influenza can be underestimated.

As far as the contribution of type of viral strain is concerned, the findings of our systematic review seem to suggest a higher mortality due to virus A, but less conclusive results may be drawn about complications and hospitalizations.

Given this, although gaps in existing data still exist, there is evidence of the significant burden that influenza places each year on the Italian population across all age groups. This is even more important considering that a projected increase of more than 30% of cases of influenza has been estimated in a 30 years' time horizon in the US adult population aged 50 years and older.^39^ Similarly, an increase in costs is forecasted and approximately 50% of productivity loss costs will be attributed to influenza‐related mortality while 75% of direct costs will be due to hospitalized cases. Indeed, the prevention of influenza is of utmost importance in particular among people with higher risk for these two outcomes. Recommendations for vaccinating high‐risk groups are already implemented in most countries and generally encompass elderly albeit with different age cut‐off,^40^ but attention should be paid also to children because mostly affected by the disease each year^41^ and at risk of complications and hospitalizations. Nevertheless, more, and much standardized data would be worthwhile to inform the decision‐making process at national level.

The findings of this systematic review should be interpreted considering the following limits. Because we restricted our review to published data available on three databases, it is not possible to exclude that we might have missed some articles. However, we believe that it is unlikely that additional relevant data could be found. Another limit is represented by the lack of pooled estimates that were not obtainable. Studies reported data across a range of seasons and settings and considered various endpoints; therefore, they used different methods for evaluating the burden of influenza. Considering this heterogeneity, a meta‐analysis of data was not performed.

This prevents having a clear estimate of probabilities of different influenza‐related complications and calls for further standardized and population‐based research in the field. Nevertheless, to the best of our knowledge, this review represents the first attempts to collect and summarize italian data and could offer clues for further research. In fact, a thorough and robust understanding of influenza‐related burden is necessary to both make health systems prepared to manage influenza cases and better exploit the potential impact of control measures, such as vaccination.

## 5 CONCLUSIONS

The evidence on influenza‐related complications, hospitalizations, and mortality in the Italian population is fragmented because of heterogeneity in study populations, settings, and methods. Nonetheless, it shows the relevant burden that influence places each year, in particular among elderly, people with underlying conditions but also children. The overview provided by our systematic review can inform the current planning of prevention measures against influenza and pinpoints areas of research that deserve further development, namely, the risk of the whole set of complications of influenza in children and high‐risk through population‐based follow up studies.

## AUTHOR CONTRIBUTIONS

**Irene Giacchetta:** Data curation; formal analysis; methodology. **Chiara Primieri:** Data curation; formal analysis; methodology. **Riccardo Cavalieri:** Data curation; formal analysis; methodology. **Alexander Domnich:** Data curation; validation. **Chiara de Waure:** Conceptualization; data curation; formal analysis; supervision; validation.

## FUNDING STATEMENT

This research received no external funding.

## ETHICS APPROVAL STATEMENT

Not applicable.

## PATIENT CONSENT STATEMENT

Not applicable.

## PERMISSION TO REPRODUCE MATERIAL FROM OTHER SOURCES

Not applicable.

### PEER REVIEW

The peer review history for this article is available at https://publons.com/publon/10.1111/irv.12925.

## Data Availability

Data sharing is not applicable to this article as no new data were created or analyzed in this study.

## Appendix A CHECK LIST PRISMA

**Table:**

Section and topic	Item #	Checklist item	Location where item is reported
**TITLE**			
Title	1	Identify the report as a systematic review.	Title
**ABSTRACT**			
Abstract	2	See the PRISMA 2020 for Abstracts checklist.	
**INTRODUCTION**			
Rationale	3	Describe the rationale for the review in the context of existing knowledge.	1.
Objectives	4	Provide an explicit statement of the objective(s) or question(s) the review addresses.	1.
**METHODS**			
Eligibility criteria	5	Specify the inclusion and exclusion criteria for the review and how studies were grouped for the syntheses.	2.1
Information sources	6	Specify all databases, registers, websites, organizations, reference lists and other sources searched or consulted to identify studies. Specify the date when each source was last searched or consulted.	2.1
Search strategy	7	Present the full search strategies for all databases, registers and websites, including any filters and limits used.	2.1
Selection process	8	Specify the methods used to decide whether a study met the inclusion criteria of the review, including how many reviewers screened each record and each report retrieved, whether they worked independently, and if applicable, details of automation tools used in the process.	2.1
Data collection process	9	Specify the methods used to collect data from reports, including how many reviewers collected data from each report, whether they worked independently, any processes for obtaining or confirming data from study investigators, and if applicable, details of automation tools used in the process.	2.1
Data items	10a	List and define all outcomes for which data were sought. Specify whether all results that were compatible with each outcome domain in each study were sought (e.g., for all measures, time points, analyses), and if not, the methods used to decide which results to collect.	2.2
	10b	List and define all other variables for which data were sought (e.g., participant and intervention characteristics, funding sources). Describe any assumptions made about any missing or unclear information.	2.2
Study risk of bias assessment	11	Specify the methods used to assess risk of bias in the included studies, including details of the tool(s) used, how many reviewers assessed each study and whether they worked independently, and if applicable, details of automation tools used in the process.	2.3
Effect measures	12	Specify for each outcome the effect measure(s) (e.g., risk ratio, mean difference) used in the synthesis or presentation of results.	N/A
Synthesis methods	13a	Describe the processes used to decide which studies were eligible for each synthesis (e.g., tabulating the study intervention characteristics and comparing against the planned groups for each synthesis [item #5]).	N/A
	13b	Describe any methods required to prepare the data for presentation or synthesis, such as handling of missing summary statistics, or data conversions.	N/A
	13c	Describe any methods used to tabulate or visually display results of individual studies and syntheses.	N/A
	13d	Describe any methods used to synthesize results and provide a rationale for the choice(s). If meta‐analysis was performed, describe the model(s), method(s) to identify the presence and extent of statistical heterogeneity, and software package(s) used.	N/A
	13e	Describe any methods used to explore possible causes of heterogeneity among study results (e.g., subgroup analysis, meta‐regression).	N/A
	13f	Describe any sensitivity analyses conducted to assess robustness of the synthesized results.	N/A
Reporting bias assessment	14	Describe any methods used to assess risk of bias due to missing results in a synthesis (arising from reporting biases).	N/A
Certainty assessment	15	Describe any methods used to assess certainty (or confidence) in the body of evidence for an outcome.	N/A
**RESULTS**			
Study selection	16a	Describe the results of the search and selection process, from the number of records identified in the search to the number of studies included in the review, ideally using a flow diagram.	3.
	16b	Cite studies that might appear to meet the inclusion criteria, but which were excluded, and explain why they were excluded.	3.
Study characteristics	17	Cite each included study and present its characteristics.	3.1, Table 1
Risk of bias in studies	18	Present assessments of risk of bias for each included study.	Table 2
Results of individual studies	19	For all outcomes, present, for each study: (a) summary statistics for each group (where appropriate) and (b) an effect estimate and its precision (e.g., confidence/credible interval), ideally using structured tables or plots.	Table 1, Table 3
Results of syntheses	20a	For each synthesis, briefly summaries the characteristics and risk of bias among contributing studies.	3.2, 3.3
	20b	Present results of all statistical syntheses conducted. If meta‐analysis was done, present for each the summary estimate and its precision (e.g., confidence/credible interval) and measures of statistical heterogeneity. If comparing groups, describe the direction of the effect.	N/A
	20c	Present results of all investigations of possible causes of heterogeneity among study results.	N/A
	20d	Present results of all sensitivity analyses conducted to assess the robustness of the synthesized results.	N/A
Reporting biases	21	Present assessments of risk of bias due to missing results (arising from reporting biases) for each synthesis assessed.	N/A
Certainty of evidence	22	Present assessments of certainty (or confidence) in the body of evidence for each outcome assessed.	N/A
**DISCUSSION**			
Discussion	23a	Provide a general interpretation of the results in the context of other evidence.	4.
	23b	Discuss any limitations of the evidence included in the review.	4.
	23c	Discuss any limitations of the review processes used.	4.
	23d	Discuss implications of the results for practice, policy, and future research.	4.
**OTHER INFORMATION**			
Registration and protocol	24a	Provide registration information for the review, including register name and registration number, or state that the review was not registered.	2.
	24b	Indicate where the review protocol can be accessed, or state that a protocol was not prepared.	N/A
	24c	Describe and explain any amendments to information provided at registration or in the protocol.	N/A
Support	25	Describe sources of financial or non‐financial support for the review, and the role of the funders or sponsors in the review.	Funding statement
Competing interests	26	Declare any competing interests of review authors.	Conflict of Interest
Availability of data, code and other materials	27	Report which of the following are publicly available and where they can be found: template data collection forms; data extracted from included studies; data used for all analyses; analytic code; any other materials used in the review.	N/A

**Table:**

Section and topic	Item #	Checklist item	Reported (yes/no)
**TITLE**			
Title	1	Identify the report as a systematic review.	Yes
**BACKGROUND**			
Objectives	2	Provide an explicit statement of the main objective(s) or question(s) the review addresses.	Yes
**METHODS**			
Eligibility criteria	3	Specify the inclusion and exclusion criteria for the review.	Yes
Information sources	4	Specify the information sources (e.g., databases, registers) used to identify studies and the date when each was last searched.	Yes
Risk of bias	5	Specify the methods used to assess risk of bias in the included studies.	Yes
Synthesis of results	6	Specify the methods used to present and synthesize results.	Yes
**RESULTS**			
Included studies	7	Give the total number of included studies and participants and summaries relevant characteristics of studies.	Yes
Synthesis of results	8	Present results for main outcomes, preferably indicating the number of included studies and participants for each. If meta‐analysis was done, report the summary estimate and confidence/credible interval. If comparing groups, indicate the direction of the effect (i.e. which group is favored).	Yes
**DISCUSSION**			
Limitations of evidence	9	Provide a brief summary of the limitations of the evidence included in the review (e.g., study risk of bias, inconsistency and imprecision).	Yes
Interpretation	10	Provide a general interpretation of the results and important implications.	Yes
**OTHER**			
Funding	11	Specify the primary source of funding for the review.	Yes
Registration	12	Provide the register name and registration number.	Yes
